# A questionnaire based study about knowledge and management of dentin hypersensitivity among dentists

**DOI:** 10.6026/973206300210533

**Published:** 2025-03-31

**Authors:** Chacko Lisa, Jaindesarda Ekata, Dighe Anisha, Kshirsagar Maithili, Barse Payal, Hitendra R. Jain

**Affiliations:** 1 Department of Periodontics, SMBT Dental College & Hospital, Sangamner, Maharashtra, India; 2 Department of Oral Medicine and Radiology, SMBT Dental College & Hospital, Sangamner, Maharashtra, India; 3 Department of Orthodontics & Dentofacial orthopaedics, SMBT Dental College & Hospital, Sangamner, Maharashtra, India; 4 Department of Public Health Dentistry, SMBT Dental College & Hospital, Sangamner, Maharashtra, India

**Keywords:** Dentin hypersensitivity, dentists, questionnaire

## Abstract

Dentin hypersensitivity, a condition that is potentially widespread and under-diagnosed, there are several various approaches available for treatment. Dentists must have a thorough understanding of dentin hypersensitivity to effectively manage it and enhance patient satisfaction and quality of life. This research aimed to evaluate the Maharashtra dental practitioners' knowledge and understanding of treating dentin hypersensitivity. A questionnaire survey based on previously conducted studies & distributed to Maharashtra's dentists. To gauge their understanding of the prevalence, etiological variables, preferred diagnosis methods and management of dentin hypersensitivity, questions were asked to the dentists. The results of the research indicate that there is still confusion about certain aspects of the diagnosis and treatment of dentin hypersensitivity among & surveyed dentist lacks awareness of the use of newer modalities for the treatment of dentin hypersensitivity.

## Background:

Multiple terms are used to describe dentin hypersensitivity (DH), depending on the location of the hypersensitivity: dentin, cemental, cervical and the words hypersensitivity and sensitivity are also used alternatively [1, 2-3]. In dentin hypersensitivity, when dentin is exposed to external stimuli- whether osmotic, tactile, evaporative, thermal, or chemical - without any associated tooth defect or disease, a short, intense pain is experienced [4]. The prevalence of dentin hypersensitivity varies widely, ranging between 1.1% to 98% in several publish study groups [5, 6, 7, 8, 9- 10]. Studies using questionnaires to evaluate patient complaints concerning dentin hypersensitivity have found a prevalence of up to 57% [11, 12, 13-14]. However, research using questionnaires to gather data from dentists has found that only 10% to 25% of cases were reported [15, 16, 17, 18, 19-20]. This demonstrates a basic discrepancy in how patients and dentists view dentin hypersensitivity, which could potentially affect patient's quality of life [21]. This study aimed to describe dentist's perception of their knowledge and management of dentin hypersensitivity, as observed in a sample of private dental practitioners in Maharashtra.

## Methods:

This study utilized a cross-sectional web-based questionnaire-based design to assess the knowledge and understanding of dental professionals practicing in Maharashtra. A 15-point questionnaire was prepared from a validated questionnaire used on the population of the UK [16, 22 and 23]. Nigeria, Brazil, India and Kuwait associated with the understanding of dentin hypersensitivity [24, 25, 26-27]. The questionnaire was designed based on worldwide reports about dentin hypersensitivity the questionnaire utilized multiple-choice questions and closed-ended questions. The questionnaire was hosted on a web-based platform, such as Google Forms, to facilitate data collection. The link to the questionnaire was distributed to potential participants via email, professional networks, or social media platforms. Participants were provided with clear instructions on how to complete the questionnaire. The study was conducted from February 2023 to April 2023 with a calculated sample size of 320 eligible dentists. Data entire were done in Microsoft Office Excel 2010 and analyses of results were done using Statistical product and service solution (SPSS) version 22 software. Descriptive statistics such as frequency and percentage were used. The p value was fixed at 0.05. Chi square test was used for comparison between qualitative parameters.

## Results:

In this survey, 55% of dentists (n = 176) frequently encountered patients with dentin hypersensitivity Figure 1(see PDF), with 57.5% reporting equal prevalence across genders (n=92) Figure 2 (see PDF). Older individuals (47.5%, n=158) sought treatment more often than younger ones (24.5%% n=78) Figure 3 (see PDF). Diagnostic aids, including case history and radiographs, were used by 68.7% of dentists, while 31.3% did not utilize them appropriately Figure 4 (see PDF). Molars (40%) were the most commonly affected teeth, followed by incisors (28%), premolars (17%) and canines (15%) Figure 5 (see PDF). Pain severity was assessed using VAS (44.5%) and VDS (43.4%) scales, with 3% not using any standard scales. Around 40% of dentists identified abrasion, abfraction, erosion and periodontal procedures as common causes of dentin hypersensitivity, but only 56.27% could differentially diagnose dentin hypersensitivity from similar conditions. While 79.3% considered clinical factors like tissue assessment and history in diagnosis Figure 6 (see PDF), only 37.5% used a combined approach (cements, lasers and desensitizing agents), with 56.3% relying solely on desensitizing agents Figure 7 (see PDF). Herbal agents were recommended by 12.5% and 37.57% suggested lasers and nanotechnology. Soft-bristled toothbrushes were advised by 49.3% and 54.3% considered endodontic treatment as a last resort for persistent pain Figure 8 (see PDF). Confidence in managing dentin hypersensitivity was reported by 54.3% of dentists, with 10% feeling very confident.

## Discussion:

Awareness of dentin hypersensitivity as a global issue affecting quality of life is growing, emphasizing the need for improved diagnosis and management. The 2003 Consensus document on dentin hypersensitivity highlights several concerns related to the effective diagnosis and management of the dentin hypersensitivity [4]. Previous studies indicating dentists often face uncertainty regarding its etiology, diagnosis and treatment [15, 16, 27 and 29]. The questionnaire utilized in this study was adapted from a validated questionnaire previously used in the UK, Kuwait, India and Brazil [16, 22, 23, 24, 25, 26- 27] which assessed the understanding of dentin hypersensitivity. In the present study, it was noted that dentin hypersensitivity was a highly prevalent complaint among patients reporting to the dental clinic. This can be influenced by factors such as population demographics, age, oral hygiene practices, dietary habits, the presence of dental conditions like tooth decay or gum disease, any deleterious habits and geographic location. Research indicates that dentin hypersensitivity can affect anywhere from 1.3% to 92.1% globally [30]. The majority, approximately 57.5% of surveyed dentists reported that they observed an equal prevalence of dentin hypersensitivity among both males & females. The literature suggests a relatively higher prevalence of dentin hypersensitivity cases among females compared to males, although this prevalence may vary between different populations [4, 28]. The surveyed dentists observed molars to be frequently affected by dentin hypersensitivity. Teeth that are frequently affected by dentin hypersensitivity can vary depending on individual cases and specific patient populations. However, some general trends have been observed in the literature. The teeth most commonly affected by dentin hypersensitivity are the canines and first premolars, followed by the incisors, second premolars and molars [31]. The tooth surfaces impacted by dentin hypersensitivity, in descending order, are the buccal surfaces, then the labial, occlusal, distal and lingual surfaces, with the incisal and palatal surfaces being the least affected [18, 32]. Age is a significant factor in the prevalence of dentin hypersensitivity, with treatment-seeking behavior varying across different age groups. In this study, dentists reported that older individuals often sought treatment. However, dentin hypersensitivity is most prevalent in individuals in their thirties and forties. Another study found that the overall prevalence of dentin hypersensitivity averages around 57%, peaking between the ages of 20 to 40 [33]. Only 68.7% of surveyed dentists reported using all diagnostic aids, which typically include a combination of patient history, clinical examination and various diagnostic tools. The specific aids used may vary depending on the dental professional's preference and available resources.

Diagnostic tests like percussion, palpation, vitality testing, trans-illumination and radiographic examination are employed to rule out other causes of dental pain [34]. Assessing the severity of dentin hypersensitivity is an important step in diagnosing and managing the condition. Various scales can be used to measure the intensity or severity of dentin hypersensitivity [35]. It's important to note that the choice of scale may vary among dental professionals and research studies. The specific scale used may depend on factors such as the patient population, the clinician's preference and the purpose of the assessment. Some commonly used scales are Verbal Analogue Scale (VAS), Visual Descriptive Scale (VDS), Non-Verbal Pain Scale (NVPS), *etc.*, Verbal Analogue Scale is most commonly used among the 45.5% of surveyed dentists. Various non-carious cervical lesions such as erosion, attrition, abrasion and certain dental procedures, such as periodontal surgical or nonsurgical interventions, such as scaling and root planing, have been associated with dentin hypersensitivity as predisposing factors, with varying prevalence rates reported in relation to non-carious cervical lesions (NCCLs) [36]. Only 40% of surveyed dentists were aware of etiologic factors responsible for dentin hypersensitivity. The difficulty in diagnosing these cervical lesions may contribute to the discrepancies in the reported prevalence of dentin hypersensitivity [25]. Certain dental procedures, such as periodontal surgeries or nonsurgical interventions like scaling and root planing, can result in temporary dentin hypersensitivity which is often transient and resolves with time [3]. When diagnosing dentin hypersensitivity, caution must be taken to exclude any other condition that may clinically present similarly to dentin hypersensitivity yet require quite different treatments for similar symptoms as dentin caries, cracked tooth syndrome and post-operative sensitivity. A proper examination is required to eliminate the conditions included in the differential diagnosis of dentin hypersensitivity [28]. Only 56% of surveyed dentists appropriately understood the differential diagnosis for dentin hypersensitivity. Diagnosing and managing dentin hypersensitivity requires evaluating soft and hard tissues, dental defects, prior use of desensitizing agents and the severity of dentin hypersensitivity [34]. Surveyed dentists included all these clinical considerations while diagnosing dentin hypersensitivity. The management of dentin hypersensitivity often involves a combination of treatment modalities tailored to the patient's specific needs typically involves tailored treatments such as desensitizing agents, cements, or laser therapy, based on severity and underlying causes [37].

Common desensitizing agents include toothpastes, mouthwashes, gels, or varnishes with ingredients like potassium nitrate, fluoride, or calcium phosphate, which alleviate sensitivity by occluding dentinal tubules or reducing nerve sensitivity. A combination approach can provide enhanced effectiveness and long-term relief for patients. In this study, the surveyed dentists primarily used desensitizing agents as the treatment modality, aligning with previous findings by Benoist *et al.* [38, 39]. Studies have suggested that a combination approach is a suitable option when the severity is greater. Herbal agents may have different levels of scientific evidence supporting their use in dentin hypersensitivity treatment. Herbal toothpaste and gels are available to treat dentin hypersensitivity; a variety of herbal agents such as rhubarb, propolis, casein, *etc.* have been evaluated as desensitizing agents for the treatment of dentin hypersensitivity [40]. Active management of DH should involve a triple C, or 3C's approach (continue, change, & cease), at the recall visit of the patient after active intervention for DH [28]. In the present study majority of surveyed dentists did not recommend herbal agents as desensitizing agents. Dental professionals can provide guidance based on the most up-to-date scientific literature and their clinical expertise. Dental cement, such as glass ionomer cement (GIC) or resin-modified glass ionomer cement can be used to seal exposed dentinal tubules and provide relief from dentin hypersensitivity. These cements help block the tubules and reduce the transmission of stimuli that cause sensitivity. Advanced treatments like laser therapy and nanotechnology are used to manage dentin hypersensitivity [41, 42]. Lasers (*e.g.*, Er:YAG, Nd:YAG, diode) reduce sensitivity by sealing dentinal tubules, while nanomaterials, incorporated into products like toothpaste and varnishes, occlude tubules and provide long-lasting relief. In this survey, only 37.5% of dentists recommended these approaches for dentin hypersensitivity management. Approximately 50% of surveyed dentists suggest soft-bristled toothbrushes in patients with dentin hypersensitivity. Patients should be advised on correct tooth brushing techniques, including the use of soft-bristle brushes and nonabrasive toothpastes, with a vertical sweeping motion to minimize damage to both hard and soft dental tissues [43]. The management of dentin hypersensitivity typically involves non-invasive approaches. Decisions regarding extraction, endodontic treatment, or periodontal surgery should be individualized based on the patient's condition and symptom severity. Root canal therapy may be suitable for patients unresponsive to occlusive or restorative treatments [44]. Only 54.3% of surveyed dentists considered endodontic treatment a last resort for dentin hypersensitivity. In the present study dental professional's confidence in managing dentin hypersensitivity was 54-64%. This may fluctuate based on their training, experience and exposure to various cases. As it also depends on the knowledge they received from professional development and research activities. In the present study dental professional's confidence in managing DH was 54-64%. This may vary with training, experience and case exposure, as well as professional development and research [45]. Providing strong theoretical and clinical training for students and continued education for young doctors is essential [46, 47].

## Conclusion:

Dentin hypersensitivity poses a significant challenge in dental practice. A study in Maharashtra revealed knowledge gaps among dental professionals, particularly in advanced treatments for Dentinal hypersensitivity. Enhancing education and awareness is crucial to improve patient care and outcomes.
